# Higher-order thermal modeling and computational analysis of laser ablation in anisotropic cardiac tissue

**DOI:** 10.1007/s10237-025-01926-x

**Published:** 2025-02-24

**Authors:** Federica Bianconi, Massimiliano Leoni, Argyrios Petras, Emiliano Schena, Luca Gerardo-Giorda, Alessio Gizzi

**Affiliations:** 1 Research Unit of Theoretical and Computational Biomechanics, Department of Engineering, Universitá Campus Bio-Medico di Roma, Via Álvaro del Portillo 21, 00128 Rome, Italy; 2 Research Unit of Measurements and Biomedical Instrumentation, Department of Engineering, Universitá Campus Bio-Medico di Roma, Via Álvaro del Portillo 21, 00128 Rome, Italy; 3 Proxima Fusion GmbH, Flößergasse 2, 81369 Munich, Germany; 4 Johann Radon Institute for Computational and Applied Mathematics (RICAM), Austrian Academy of Sciences, Altenbergerstrasse 69, Linz, 4040 Austria; 5 Fondazione Policlinico Universitario Campus Bio-Medico, Via Álvaro del Portillo 200, 00128 Rome, Italy; 6 Institute for Mathematical Methods in Medicine and Data Based Modeling, Johannes Kepler University, Altenbergerstrasse 69, Linz, A-4040 Austria

**Keywords:** Higher-order thermal formulation, Finite element, Computational modeling, Laser ablation

## Abstract

Laser ablation techniques employ fast hyperthermia mechanisms for diseased-tissue removal, characterized by high selectivity, thus preserving the surrounding healthy tissue. The associated modeling approaches are based on classical Fourier-type laws, though a limited predictivity is observed, particularly at fast time scales. Moreover, limited knowledge is available for cardiac tissue compared to radiofrequency approaches. The present work proposes a comprehensive modeling approach for the computational investigation of the key factors involved in laser-based techniques and assessing the outcomes of induced cellular thermal damage in the cardiac context. The study encompasses a comparative finite element study involving various thermal and cellular damage models incorporating optical–thermal couplings, three-state cellular death dynamics, and a second-order heat transfer formulation generalizing the classical Fourier-based heat equation. A parametric investigation of the thermal profiles shows that higher-order models accurately capture temperature dynamics and lesion formation compared with the classical Fourier-based model. The results highlight the critical role of cardiac anisotropy, influencing the shape and extent of thermal damage, while the three-state cell death model effectively describes the transition from reversible to irreversible damage. These findings demonstrate the reliability of higher-order thermal formulations, laying the basis for future investigations of arrhythmia management via in silico approaches.

## Introduction

In the landscape of thermal ablation techniques, laser ablation (LA) uses focused laser energy to selectively heat and destroy abnormal tissue through a hyperthermic mechanism (Schena et al. 2017; Mooney et al. 2017; De Vita et al. 2023). This minimally invasive approach, achieved by a quick increase in temperature above ${50}\,^{\circ }\hbox {C}$, offers several advantages over traditional open-heart surgery and radiofrequency techniques (Splinter et al. 1991). Though mainly applied for cancer treatment (Pacella et al. 2005; Silva et al. 2016; Schena and Majocchi 2014; Mooney et al. 2017), LA is eligible to treat cardiac arrhythmias by targeting ectopic foci: Laser light is converted into heat in the target volume, leading to coagulative necrosis, secondary degeneration, and atrophy (Verhey et al. 2003; Poa et al. 2009; Skeete et al. 2021).

Temperature dynamics profoundly influence ablation procedure outcomes. Recently, significant effort has been devoted to accurately predict thermoelastic patterns in cardiac tissue using radiofrequency techniques (Molinari et al. 2022; Singh and Melnik 2020), thus estimating the expected size of the lesion according to the underlying microstructure. In LA, such modeling is lacking, and, in addition, the distribution and intensity of the induced thermal energy vary notably depending on the specific tissue’s optical properties, thus affecting the precision and effectiveness of the procedure. Slight deviations in temperature can lead to under or over-treatment, causing insufficient lesion formation or damage to the surrounding healthy tissue.

To address these challenges, accurate computational modeling and in silico analyses that can predict how heat propagates through tissue during LA are crucial to augment cardiac tissue treatment predictability. The present contribution fills the gap by providing a generalized computational framework comparing and contrasting higher-order thermal theories to forecast LA behavior, thus predicting accurate lesion size via an extensive numerical study. We consider a simplified three-dimensional computational domain representative of the cardiac tissue, embedding rotation anisotropy and implementing a customized finite element code solving the fully coupled optical–thermal framework, thus comparing different numerical and physical parametrizations.

Starting from the classical Pennes bioheat equation, based on Fourier theory (Pennes 1948), we then introduce higher-order models, including a generalization of the classical bioheat model (GF) and the dual-phase lag (DPL) model (Tzou 1995), to progressively account for finite heat propagation effects and microstructural interactions. Among these higher-order models, the single-phase lag (SPL) model, introduced by Cattaneo and Vernotte (Cattaneo 1958; Vernotte 1958), was also considered but not explored due to fluctuations in the thermal response not expected in soft biological tissues (Molinari et al. 2022) (see App. A). The GF model, on the other hand, provides an extended heat transfer formulation, generalizing the classical Fourier-based heat equation while maintaining physical plausibility. Furthermore, full integration with a three-state cell death model is enforced in a multiscale perspective to analyze the ablation-induced thermal damage compared to the benchmark of the ${50}\,^{\circ }\hbox {C}$ isotherm, frequently used to estimate the ablation zone boundary (Berjano 2006). A dedicated finite element implementation complements the theoretical study to solve the coupled model with high numerical accuracy.

The developed models are formulated with temperature in Kelvin; any thresholds mentioned in Celsius are provided for clarity, aligning with standard clinical practice.

## Materials and methods

The LA computational model is implemented in in-house Python code using the open-source FEniCSx library (Baratta et al. 2023; Scroggs et al. 2022; Alnæs et al. 2014). The simulated energy protocol includes a laser ablation phase of 30 s at 3 W of power delivery, followed by 270 s of relaxation period. The model incorporates the optical diffusion approximation (ODA) model to describe the laser–tissue interaction. Heat transfer in cardiac tissue is modeled via classical bioheat, a generalization of classical bioheat, and DPL models, respectively. The resulting thermal damage is reconstructed by a three-state cell model.

### Optical modeling

Optical diffusion modeling is widely used in biomedical optics to recover the mechanisms underlying light propagation within a biological medium (Tricoli et al. 2018). In this context, the ODA derives from the more comprehensive Boltzmann radiative transport equation (Tricoli et al. 2018; Reynoso et al. 2013) describing the transport of radiative energy through a medium, accounting for absorption, scattering, and emission processes (Wang and Wu 2007). Due to its intrinsic complexity, the ODA approach simplifies the modeling, thus allowing for a more manageable diffusion equation.

ODA is appropriate under specific conditions, i.e., when the scattering process dominates the absorption one, i.e., $\mu _s\gg \mu _a$, where $\mu _a$ and $\mu _s$ are the absorption and scattering coefficients of the medium, respectively. In biological tissue, especially in the near-infrared (NIR) region, this condition is typically satisfied, being $\mu _s\sim 10^2-10^3$ times greater than $\mu _a$ (Reynoso et al. 2013), justifying the use of ODA. In this context, optical properties play a crucial role influencing the distribution of laser energy within the tissue and thus small variations can lead to substantial differences in the predicted thermal behavior. Following Splinter et al. (Splinter et al. 1991), we assumed $\mu _s = {1.775 \times 10^{4}}\hbox { m}^{-1}$ and $\mu _a = {3\times 10^{1}}\hbox { m}^{-1}$ for the myocardium.

The ODA equation is defined as follows:

1 $$\begin{aligned} -D\nabla \cdot (\nabla \varphi (x,y,z)) + \mu _a \varphi (x,y,z) = s(x,y,z) \end{aligned}$$

where $\varphi (x,y,z)$ [$\hbox {W m}^{-2}$] is the light fluence rate, $\mu _a$ the absorption coefficient and *s*(*x*, *y*, *z*) [$\hbox {W m}^{-3}$] the light source term. The tissue diffusion coefficient $D={1}/{3(\mu _{s}^{'}+\mu _a)}$ [m] is derived from the material optical properties (Swartling et al. 2003), where $\mu _{s}'=(1-g)\mu _s$ [$\hbox { m}^{-1}$] is the reduced scattering coefficient and *g* the tissue anisotropy factor. The latter takes into account the effects of scattering on the light beam directions; it ranges from 0.8 to 1 for biological tissues (Reynoso et al. 2013). A summary of the optical properties considered in the numerical analysis is provided in Table 1.

For the optical modeling, a pivotal assumption is made on the laser source term, directed in the *z*-direction, being the laser a strongly collimated light. With this assumption, (1) reads:

2 $$\begin{aligned} -D\frac{\partial ^2 \varphi (z)}{\partial z^2} + \mu _a \varphi (z) = s(z) \end{aligned}$$

where the laser lateral spread is approximated by a Gaussian distribution. Accordingly, a custom bidimensional Gaussian shape is applied to the laser applicator surface:

3 $$\begin{aligned} I=\frac{P}{2\pi \sigma ^2}e^{-\frac{x^{2}+y^{2}}{2\sigma ^2}} \end{aligned}$$

where P [ W] is the output laser power, $\sigma =r_{f}/3$ the standard deviation, and $r_{f}={0.4}\,\hbox {mm}$ the laser applicator radius.

### Thermal modeling

In this section, we explore three distinct heat transfer models reproducing the thermal spatiotemporal dynamics in the LA procedure. The discussion begins with the classic Fourier-based bioheat model, considered the reference framework for heat conduction in biological tissue. Afterward, we delve into a generalization of the classical bioheat model (GF), incorporating an intermediate level of complexity to bridge the gap between higher-order formulations. In particular, our exploration extends to the dual-phase lag model, a sophisticated approach to recognizing the intricate nature of energy propagation within tissue.

### Fourier-based bioheat model

The classic bioheat model is based on the Fourier law (Lopez Molina et al. 2014), assuming heat flux, $\textbf{q}$, proportional to the negative gradient of the temperature field, $\textbf{q}=-\textbf{k} \nabla \textrm{T}$, where $\textbf{k}$ [$\hbox {W m}^{-1}\,\hbox {K}^{-1}$] is the thermal conductivity tensor and $\nabla$ the gradient operator in Cartesian coordinates. Specifically, Pennes bioheat model, introduced in 1948 (Pennes 1948), reads:

4 $$\begin{aligned} {\rho } \mathrm {c(T)}\frac{\partial \textrm{T}}{\partial t} = \nabla \cdot (\textbf{k}\mathrm {(T)} \nabla \textrm{T}) + \textrm{Q}_{\textrm{b}} + \textrm{Q}_{\textrm{m}} + \textrm{Q}_{\textrm{s}} \end{aligned}$$

where $\textrm{T}$ [K] is the tissue temperature, ${\rho }$ [$\hbox {kg m}^{-3}$] the density, $\mathrm {c(T)}$ [$\hbox {J}\,\hbox {Kg}^{-1}\,\hbox {K}^{-1}$] the heat capacity, and $\textrm{Q}_{\textrm{b}}=-\rho _{b} \textrm{c}_{\textrm{b}} \omega _{\textrm{b}}(\textrm{T}-\textrm{T}_{\textrm{b}})$, $\textrm{Q}_{\textrm{m}}$, $\textrm{Q}_{\textrm{s}}$ = $\mu _a \varphi$ [$\hbox {W m}^{-3}$] are the blood perfusion, metabolic heat, and laser heating source contributions, respectively.

The local high increase in tissue temperature due to LA procedure further motivates the adoption of a generalized framework. We assume, in particular, temperature dependency for the myocardial constitutive parameters $\mathrm {c(T)}$ and $\textbf{k}\mathrm {(T)}$ (Petras et al. 2019). Specifically, a linear decreasing relation is considered for the heat capacity:

5 $$\begin{aligned} \mathrm {c(T)}=\textrm{c}_{\textrm{0}}(1+\textrm{c}_{\textrm{1}}(\mathrm {T-T}_{\textrm{myo}})) \end{aligned}$$

where $\textrm{c}_{\textrm{0}}$ is the reference value at body temperature, $\textrm{c}_{\textrm{1}}$ is a non-dimensional constant gain and $\textrm{T}_{\textrm{myo}} = {37}\,^{\circ }\hbox {C}$ is the reference body temperature.

Besides, the thermal conductivity tensor $\textbf{k}\mathrm {(T)}$ is characterized to reproduce anisotropic thermal diffusivity, typical of myocardial microstructure:

6 $$\begin{aligned} \textbf{k}({\textrm{T}})={\textbf{R}} \begin{bmatrix} \textrm{k}_{\textrm{f}}(\textrm{T}) & 0 & 0 \\ 0 & \textrm{k}_{\textrm{t}}(\textrm{T}) & 0 \\ 0 & 0 & \textrm{k}_{\textrm{n}}(\textrm{T}) \end{bmatrix}\textbf{R}^{\textbf{T}} \end{aligned}$$

where $\textrm{k}_{i}(\textrm{T})$, with $i=\textrm{f,t,n}$, represent the temperature-dependent thermal conductivities in the fiber, transverse, and normal directions, respectively, following a linear dependency:

7 $$\begin{aligned} \textrm{k}_i( \textrm{T})=\textrm{k}_0(1+\textrm{k}_1(\textrm{T}-\textrm{T}_{\textrm{myo}})) \end{aligned}$$

where $\textrm{k}_{\textrm{0}}$ is the thermal conductivity at body temperature, and $\textrm{k}_{\textrm{1}}$ a non-dimensional constant gain. Additionally, in the analysis we assume transversal conductivities equal and proportional to $\textrm{k}_{\textrm{f}}$. Specifically, $\textrm{k}_{\textrm{t}} = \textrm{k}_{\textrm{n}} = \textrm{k}_{\textrm{f}}/\textrm{k}_{\textrm{ratio}}$, whereby $\textrm{k}_{\textrm{ratio}}$ is the anisotropy ratio, object of an upcoming computational parametric analysis.

In (6), ${\textbf{R}}$ represents the rotation matrix, based on the local fiber reference system:

8 $$\begin{aligned} {\textbf{R}}=\begin{bmatrix} \cos \theta & -\sin \theta & 0\\ \sin \theta & \cos \theta & 0\\ 0& 0& 1 \end{bmatrix} \end{aligned}$$

Following Molinari et al. (Molinari et al. 2022), we considered a rotational anisotropy in the ventricular myocardium. This involves a ${120}^{\circ }$ counterclockwise rotation of the fibers from the outermost (epicardium) to the innermost layer (endocardium) (Lombaert et al. 2012). The myocardial fibers rotate across the ventricular wall, creating a sheet-like transversely isotropic material that alters the conductivity properties throughout the depth. Thus, the material is characterized by both directional dependence (anisotropy) and non-uniformity (heterogeneity). The fibers’ rotational anisotropy is mathematically described as:

9 $$\begin{aligned} \theta (\textrm{z})= \theta _{\textrm{epi}}+\frac{\mathrm {z-z}_{\textrm{epi}}}{\textrm{z}_{\textrm{endo}}-{\textrm{z}}_{\textrm{epi}}} (\theta _{\textrm{endo}}-\theta _{\textrm{epi}}) \end{aligned}$$

where z is the thickness direction.

### Generalized Fourier model

The Fourier model assumes an instantaneous response to thermal energy input, meaning that any temperature change is immediately reflected throughout the medium. This assumption simplifies the analysis but may not always capture the transient nature of heat transfer, especially in biological tissue where the response can be delayed. Indeed, classical Fourier-based models struggle to accurately represent the finite speed of thermal propagation, primarily due to biological tissue complexity and its heterogeneous microstructure. This limitation becomes particularly evident in hyperthermal treatments, where rapid temperature rises occur within short time frames and are driven by steep temperature gradients (Molinari et al. 2022). Such dynamic and localized thermal behavior demands a more advanced approach to fully capture the underlying physics.

Different theories have been proposed to account for non-Fourier behavior, including hyperbolic heat equations and relativistic heat transfer. The scientific literature indicates that thermal behavior in non-homogeneous media requires a relaxation time to accumulate enough energy before transferring it to the nearest element. In this work, we first introduce a generalization of the Fourier-based model (GF) by incorporating a pseudo-convective term in (4), governed by the time constant $\tau _{t}$. The modified equation is expressed as follows:

10 $$\begin{aligned} {\rho } \mathrm {c(T)}\frac{\partial \textrm{T}}{\partial t} = \nabla \cdot \left( \textbf{k}\mathrm {(T)} \nabla \textrm{T} \right) + \tau _{t} \nabla \cdot \left( \textbf{k}\mathrm {(T)} \frac{\partial \nabla \textrm{T}}{\partial t} \right) + \textrm{Q}_{\textrm{b}} + \textrm{Q}_{\textrm{m}} + \textrm{Q}_{\textrm{s}} \end{aligned}$$

where $\tau _{t}$ is the phase lag of the temperature gradient. Notably, if $\tau _{t} = 0$, the equation reduces to the classical bioheat heat conduction equation, effectively removing the pseudo-convective term.

### Dual-phase lag model

Among the theories developed to account for non-Fourier behavior, Tzou (Tzou 1995) proposed a hyperbolic heat equation considering delays in both the heat flux and temperature gradient. The DPL model, initially applied to solid materials like metals under rapid heating conditions, has since been extended to biological tissues due to its ability to capture the transient thermal dynamics and heterogeneities inherent in such systems (Afrin et al. 2012; Singh and Melnik 2019). The model recognizes that in biological tissue, the propagation of thermal energy involves complex interactions at both the macroscopic and microscopic levels. Accordingly, two phase lag times are considered: $\tau _{q}$ defining the time lag between the heat flux and temperature gradient, and $\tau _{t}$ representing the phase lag in establishing the temperature gradient in the conductive medium. The DPL model is required to catch the initial transient temperature increase, which has a critical impact on the evolution of the damage pattern in the tissue. Mathematically, the DPL equation reads as a second-order generalization of (10):

11 $$\begin{aligned} \tau _{\textrm{q}} {\rho } \mathrm {c(T)} \frac{\partial ^2 \textrm{T}}{\partial \textrm{t}^{2}} + {\rho } \mathrm {c(T)}\frac{\partial \textrm{T}}{\partial t}=&\nabla \cdot (\textbf{k}\mathrm {(T)} \nabla \textrm{T}) + \tau _{t} \nabla \cdot \left( \textbf{k}\mathrm {(T)} \frac{\partial \nabla \textrm{T}}{\partial t}\right) \nonumber \\\quad &+ \textrm{Q}_{\textrm{b}} + \textrm{Q}_{\textrm{m}} + \textrm{Q}_{\textrm{s}} \end{aligned}$$

The continuous model is completed with dedicated boundary conditions that define a well-posed problem as described in Sec. 4.

### Three-state cell modeling

At the cellular level, prolonged exposure to high temperatures results in protein denaturation and irreversible damage (Pearce 2013; Liu et al. 2018). Moreover, delayed cellular death has also been observed for cells that suffered injuries beyond recovery through intrinsic mechanisms such as, among others, apoptosis and necroptosis. Several models have been introduced to assess the irreversible cellular damage and estimate the resulting lesion size. Irreversible damage is expected for temperatures above ${50}\,^{\circ }\hbox {C}$; thus, the most straightforward approach uses the ${50}\,^{\circ }\hbox {C}$ isotherm contour to identify the lesion boundary. This approach typically overestimates the myocardial lesion size and does not allow a physiologically accurate assessment of the lesion (Liu et al. 2018; Haines 2011).

Another approach uses a two-state Arrhenius model to describe the transition of the cells from native to denatured state (Feng et al. 2008). Despite being widely used, it is well known that this model dramatically overpredicts the shoulder region at low temperatures, where the dynamics of the thermal damage are slower (Pearce 2013; Liu et al. 2018; O’Neill et al. 2011). This shortcoming is particularly relevant for thermal damage of myocytes since it is known that at ${48}\,^{\circ }\hbox {C}$ the damage is reversible for all treatment times (Zaltieri et al. 2021). Irreversible damage is expected between ${50} \div {56}\,^{\circ }\hbox {C}$ for up to 60 s of ablation (Haines 2011).

In this context, the three-state thermal cell model initially introduced by Park et al. (Park et al. 2016) can effectively represent the reversible cell damage, making it a valuable tool for estimating lesions in ablation models. The three-state model consists of three cellular states: the native (N) state, representing a normally functional cell, the unfolded (U) state for cells that suffered some damage that might be reversible, and the denatured (D) state, where the cell is irreversibly damaged. The interaction of the three states can be summarized as follows:

12 $$\begin{aligned} \textrm{N} \underset{\alpha _{3}\mathrm {(T)}}{{\mathop {\rightleftharpoons }\limits ^{\alpha _{1}\mathrm {(T)}}}} \textrm{U} {\mathop {\longrightarrow }\limits ^{\alpha _{2}\mathrm {(T)}}} \textrm{D} \end{aligned}$$

where ${\alpha }_{i}(\textrm{T})$, with $i = 1, 2, 3$, are temperature-dependent transition rates governed by the following system of ordinary differential equations:

13 $$\begin{aligned} {\left\{ \begin{array}{ll} \begin{aligned} & \frac{d \textrm{N}}{dt}=-\alpha _{1}\mathrm {(T)} \textrm{N}+\alpha _{3}\mathrm {(T)} \textrm{U} \\ & \frac{d \textrm{U}}{d t}= \quad \alpha _{1}\mathrm {(T)} \textrm{N}-\left( \alpha _{2}\mathrm {(T)}+\alpha _{3}\mathrm {(T)}\right) \textrm{U} \\& \frac{d \textrm{D}}{d t}= \quad \alpha _{2}\mathrm {(T)} \textrm{U} \end{aligned} \end{array}\right. } \end{aligned}$$

The system is initialized by considering all cells in the native state, namely $\textrm{N}$ = 1, $\textrm{U}$ = 0, and $\textrm{D}$ = 0. Due to the time scale of the ablation process (of the order of minutes), no cell proliferation is considered in the model (of the order of months), and a conservation equation holds at all times for the three states ($\textrm{N}$ + $\textrm{U}$ + $\textrm{D}$ = 1) (He and Zhou 2017). In the original formulation of Park et al. (Park et al. 2016), the transitions among the phases are modeled using an Arrhenius model: ${\alpha }_{i}\mathrm {(T)} = \textrm{A}_{i}e^{-\Delta \textrm{E}_{i} / \mathrm {(RT)}}$, $i = 1, 2, 3$, where $\textrm{A}_i$ is the frequency factor [$\hbox {s}^{-1}$], $\Delta \textrm{E}_{i}$ is the activation energy [$\hbox {J mol}^{-1}$], and R is the universal gas constant.

Nevertheless, the model is not specific to myocytes. To overcome this limitation, we follow Petras et al. (Petras et al. 2023) with a modification of the three-state model, which accounts for the variable energy absorption rate and is calibrated for the cardiac tissue. In particular, the transition phases are modified using a piecewise Arrhenius model that changes slope at ${55}\,^{\circ }\hbox {C}$, as evidenced by experiments (Qin et al. 2014). The transition rate $\alpha _{1}$ exhibits a slope change at ${55}\,^{\circ }\hbox {C}$, reflecting the regime change in absorption rates. The overall frequency factor and activation energy are given as $\textrm{A} = {\hbox {A}_{1} \hbox {A}_{2}/\hbox {A}_{3}}$ and $\Delta \textrm{E} = \Delta \textrm{E}_{\textrm{1}} + \Delta \textrm{E}_{\textrm{2}} - \Delta \textrm{E}_{3}$, respectively. For $\textrm{T} \le {55}\,^{\circ }\hbox {C}$, $\textrm{A} = {2.97\times 10^{70}}\hbox {s}^{-1}$ and $\Delta \textrm{E} = {4.484 \times 10^{5}}\hbox {J mol}^{-1}$. In the case $\textrm{T} > {55}\,^{\circ }\hbox {C}$, the overall frequency factor and activation energy become $\textrm{A} = {1.19\times 10^{19}}\hbox {s}^{-1}$ and $\Delta \textrm{E} = {1.255\times 10^{5}}\hbox {J mol}^{-1}$, respectively.

Finally, considering the several intrinsic mechanisms contributing to the slow death of a damaged cell, we use a threshold value for the cells in the native state N, below which cell death is expected to occur. Since no specific data are available for cardiac myocytes, we consider the irreversible damage threshold to be N $\le 0.8$ following Petras et al. (Petras et al. 2019).

**Table 1:** Optical, thermal, and cell death model parameters

Parameter		Value	[u]
Optical Model			
Myocardium absorption coefficient	$\mu _a$	${3\times 10^{1}}$	$\hbox { m}^{-1}$
Myocardium scattering coefficient	$\mu _s$	${1.775\times 10^{4}}$	$\hbox { m}^{-1}$
Myocardium anisotropy factor	*g*	0.964	
Thermal Model			
Myocardium density	$\rho$	1076	$\hbox {kg m}^{-3}$
Reference thermal conductivity	$\textrm{k}_{0}$	0.518	$\hbox {W m}^{-1}\,\hbox {K}^{-1}$
Thermal conductivity constant	$\textrm{k}_{{1}}$	$-$0.0005	$\hbox {K}^{-1}$
Reference heat transfer coefficient	$\textrm{c}_{{0}}$	3017	$\hbox {J Kg}^{-1}\hbox { K}^{-1}$
Heat transfer constant	$\textrm{c}_{{1}}$	$-$0.0042	$\hbox {K}^{-1}$
Blood density	$\rho _{b}$	1050	$\hbox {kg m}^{-3}$
Blood perfusion rate	$\omega _{\textrm{b}}$	0.0371	$\hbox {s}^{-1}$
Blood specific heat	$\textrm{c}_{\textrm{b}}$	3617	$\hbox {J Kg}^{-1}\hbox { K}^{-1}$
Metabolic term	$\textrm{Q}_{\textrm{m}}$	33800	$\hbox {W m}^{-3}$
Cell death Model			
Frequency factor N $\longrightarrow$ U ($\textrm{T}\le$ ${55}\,^{\circ }\hbox {C}$)	$\hbox {A}_{1}$	$8.87\times 10^{73}$	$\hbox {s}^{-1}$
Frequency factor N $\longrightarrow$ U ($\textrm{T} >$ ${55}\,^{\circ }\hbox {C}$)	$\hbox {A}_{1}$	${3.56\times 10^{22}}$	$\hbox {s}^{-1}$
Frequency factor U $\longrightarrow$ D	$\hbox {A}_{2}$	${5.35\times 10^{11}}$	$\hbox {s}^{-1}$
Frequency factor U $\longrightarrow$ N	$\hbox {A}_{3}$	${1.6\times 10^{15}}$	$\hbox {s}^{-1}$
Activation energy N $\longrightarrow$ U ($\textrm{T}\le$ ${55}\,^{\circ }\hbox {C}$)	$\Delta \textrm{A}_{{1}}$	$4.676\times 10^{5}$	$\hbox {J mol}^{-1}$
Activation energy N $\longrightarrow$ U ($\textrm{T} >$ ${55}\,^{\circ }\hbox {C}$)	$\Delta \textrm{A}_{{1}}$	${1.447\times 10^{5}}$	$\hbox {J mol}^{-1}$
Activation energy U $\longrightarrow$ D	$\Delta \textrm{A}_{2}$	${8.59\times 10^{4}}$	$\hbox {J mol}^{-1}$
Activation energy U $\longrightarrow$ N	$\Delta \textrm{A}_{3}$	${1.051\times 10^{5}}$	$\hbox {J mol}^{-1}$

## Weak formulation

**Fig. 1:**
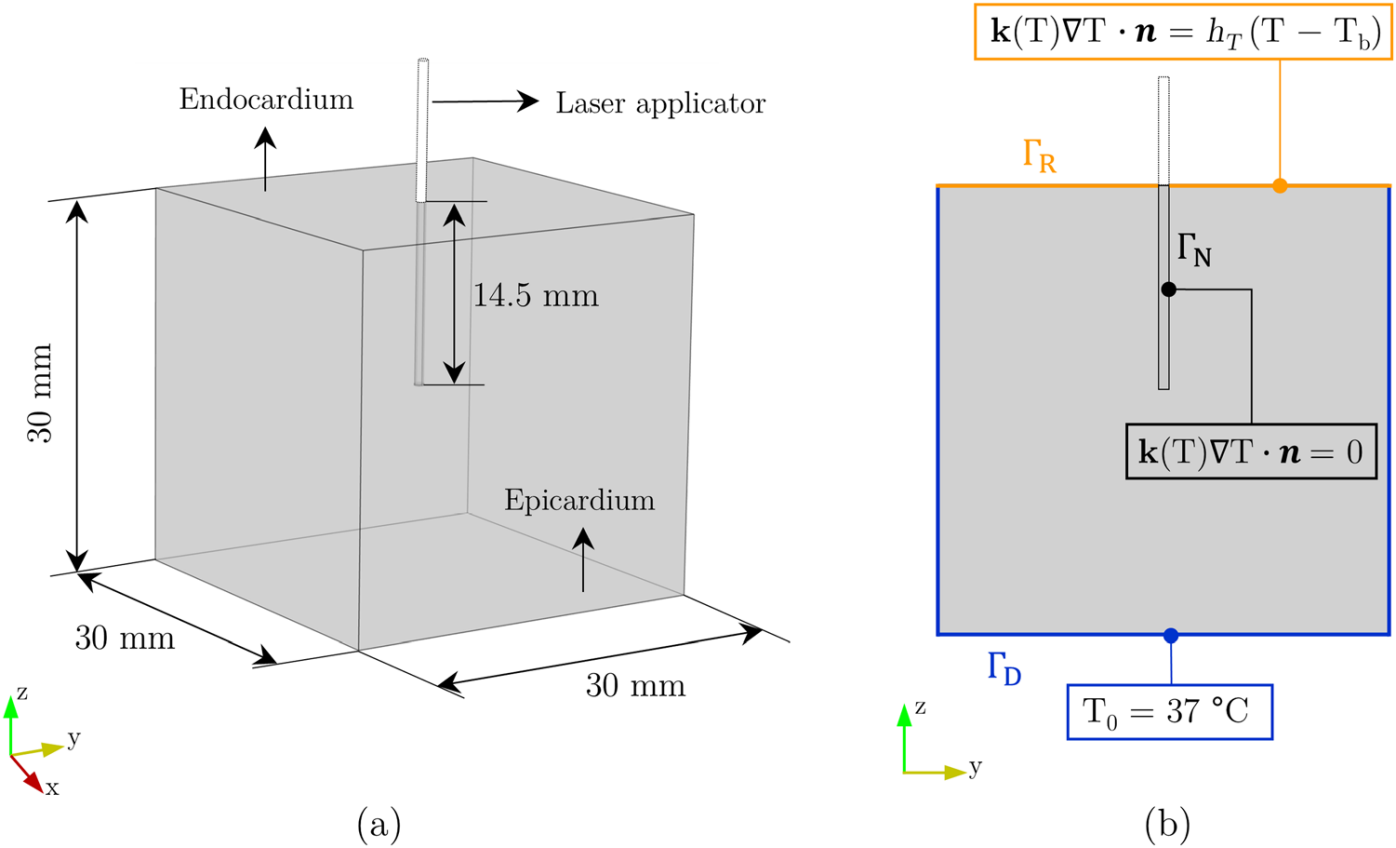
**a** Computational domain: cube of side 30 mm. The laser applicator is inserted for 14.5 mm within the domain. **b** Thermal boundary conditions. Thermal insulation is considered at the boundary between the laser catheter and the myocardium tissue. A Robin boundary condition is imposed on the endocardial surface in contact with the blood. A Dirichlet boundary condition is enforced to the surrounding boundaries at $\textrm{T}_{{0}}$ = ${37}\,^{\circ }\hbox {C}$

The nonlinear models illustrated in the previous sections are discretized in time via an implicit Euler scheme, linearized around the previous time step, and approximated via a custom finite element solver. In detail, the three bioheat models (4), (10), (11) describe a non-steady problem in the variable T, whose boundary is composed as $\partial \Omega = \Gamma _{\textrm{R}} + \Gamma _{\textrm{D}} + \Gamma _{\textrm{N}}$, where $\Gamma _{\textrm{R}}$, $\Gamma _{\textrm{D}},$ and $\Gamma _{\textrm{N}}$ are the Robin, Dirichlet, and Neumann boundaries, respectively (see Fig. 1b). Accordingly, naming trial function at step n+1 $\hbox {T}^{n+1}\in H^1(\Omega _D)$ and test function $\textrm{v}\in H^1(\Omega _D)$, the weak formulation of the Fourier model reads:

14 $$\begin{aligned} \begin{aligned}&\int _{\Omega } {\rho } \textrm{c}(\hbox {T}^{n}) \frac{\textrm{T}^{n+1} - \textrm{T}^{n}}{\Delta \textrm{t}} \textrm{v} \ dV \ + \ \int _{\Gamma _{\textrm{R}}} h_T (\textrm{T}^{n+1} - \textrm{T}_{\textrm{myo}})\textrm{v} \ dS \ + \\&\quad \quad + \ \int _{\Omega } \textbf{k}(\textrm{T}^{n}) \nabla \textrm{T} ^{n+1} \nabla \textrm{v} \ dV = \int _{\Omega } (\textrm{Q}_{\textrm{b}} + \textrm{Q}_{\textrm{m}} + \textrm{Q}_{\textrm{s}}) \textrm{v} \ dV \end{aligned} \end{aligned}$$

The weak formulation of the GF model is:

15 $$\begin{aligned} \begin{aligned}&\int _{\Omega } {\rho } \textrm{c}(\hbox {T}^{n}) \frac{\textrm{T}^{n+1} - \textrm{T}^{n}}{\Delta \textrm{t}} \textrm{v} \ dV + \int _{\Gamma _{\textrm{R}}} h_T(\textrm{T}^{n+1} - \textrm{T}_{\textrm{myo}})\textrm{v} \ dS \ + \\&\quad \quad + \ \int _{\Omega } \textbf{k}(\textrm{T}^{n}) \nabla \textrm{T} ^{n+1} \nabla \textrm{v} \ dV \ + \\&\quad \quad + \ \tau _{t} \int _{\Gamma _{R}} \frac{h_T\left( \textrm{T}^{n+1} - \textrm{T}_{\textrm{myo}}\right) - h_T\left( \textrm{T}^{n} - \textrm{T}_{\textrm{myo}}\right) }{\Delta \textrm{t}} \textrm{v} \ dS \ + \\&\quad \quad + \ \tau _{t} k_{0} k_{1} \int _{\partial \Omega } \textrm{v} \frac{\textrm{T}^{n+1} - \textrm{T}^{n}}{\Delta \textrm{t}} \nabla \textrm{T}^{n+1} \cdot \hat{\textbf{n}} \ dS \ + \\&\quad \quad + \ \tau _{t} \int _{\Omega } \nabla \textrm{v} \textbf{k}(\textrm{T}^{n}) \frac{\nabla \textrm{T}^{n+1} - \nabla \textrm{T}^{n}}{\Delta \textrm{t}} \ dV \\&\quad = \int _{\Omega } (\textrm{Q}_{\textrm{b}} + \textrm{Q}_{\textrm{m}} + \textrm{Q}_{\textrm{s}}) \textrm{v} \ dV \end{aligned} \end{aligned}$$

and the weak formulation of the DPL model is:

16 $$\begin{aligned} \begin{aligned}&\tau _{q} \int _{\Omega } {\rho } \textrm{c}(\hbox {T}^{n}) \frac{\textrm{T}^{n+1} - 2\textrm{T}^n + \textrm{T}^{n-1}}{\Delta \textrm{t}^2} \textrm{v} \; dV \\&\quad \quad + \int _{\Omega } {\rho } \textrm{c}(\hbox {T}^{n}) \frac{\textrm{T}^{n+1} - \textrm{T}^{n}}{\Delta \textrm{t}} \textrm{v} \; dV \;+ \\&\quad \quad + \int _{\Gamma _{\textrm{R}}} h_T(\textrm{T}^{n+1} - \textrm{T}_{\textrm{myo}})\textrm{v} \;dS\; \\&\quad \quad + \; \int _{\Omega } \textbf{k}(\textrm{T}^{n}) \nabla \textrm{T}^{n+1} \nabla \textrm{v} \;dV \; + \\&\quad \quad + \; \tau _{t}\int _{\Gamma _{\textrm{R}}} \frac{h_T \left( \textrm{T}^{n+1} - \textrm{T}_{\textrm{myo}}\right) - h_T \left( \textrm{T}^{n} - \textrm{T}_{\textrm{myo}}\right) }{\Delta \textrm{t}} \textrm{v} \;dS \\&\quad \quad + \tau _{t} k_{0} k_{1} \int _{\partial \Omega } \textrm{v} \frac{\textrm{T}^{n+1} - \textrm{T}^{n}}{\Delta \textrm{t}} \nabla \textrm{T}^{n+1} \cdot \hat{\textbf{n}} \; dS \;\\&\quad \quad + \; \tau _{t} \int _{\Omega } \nabla \textrm{v} \textbf{k}(\textrm{T}^{n}) \frac{\nabla \textrm{T}^{n+1} - \nabla \textrm{T}^{n}}{\Delta \textrm{t}} \; dV \; \\&\quad = \int _{\Omega } (\textrm{Q}_{\textrm{b}} + \textrm{Q}_{\textrm{m}} + \textrm{Q}_{\textrm{s}}) \textrm{v} \;dV \end{aligned} \end{aligned}$$

## Computational model

Figure 1a depicts the computational domain developed in the open-source software suite *SALOME* ^®^*9.11.0* (Ribes and Caremoli 2007) to solve the numerical model. As visible in the figure, the laser applicator, with radius $r_f = {0.4}\,\hbox {mm}$, is placed at the center of the domain. We omitted the complete representation of the laser applicator geometry to reduce the computational cost, applying a suitable insulating boundary condition to account for it.

Figure 1b highlights the applied boundary conditions: A constant temperature $\textrm{T}_{{0}}$ = ${37}\,^{\circ }\hbox {C}$ is imposed on the outer boundaries of the cardiac domain; thermal insulation is assumed at the catheter surface $\textbf{k}\mathrm {(T)} \nabla \textrm{T} \cdot \textbf{n}=0$; and a Robin boundary condition is imposed on the upper boundary to account for the blood stream:

17 $$\begin{aligned} {\left\{ \begin{array}{ll} \textrm{T} = \textrm{T}_{0} & \text { on }\Gamma _{\textrm{D}}\\ \textbf{k}\mathrm {(T)} \nabla \textrm{T} \cdot \textbf{n}=0 & \text { on } \Gamma _{\textrm{N}}\\ \textbf{k}\mathrm {(T)} \nabla \textrm{T} \cdot \textbf{n} = h_T (\textrm{T}-\textrm{T}_{\textrm{b}}) & \text { on }\Gamma _{\textrm{R}} \end{array}\right. } \end{aligned}$$

The thermal convective coefficient at the blood–tissue surface is calculated according to (González-Suárez and Berjano 2015):

18 $$\begin{aligned} w=\left( \frac{h_T}{h_{ref}}\right) ^{1.25} w_{ref} \end{aligned}$$

where *w* is the blood velocity in the cardiac chamber, $w_{ref}$ = ${24}\,\hbox {cm s}^{-1}$ is the reference blood velocity measured inside the cardiac chamber, and $h_{ref}$ = ${1417}\,\hbox {W m}^{-2}\hbox { K}^{-1}$ is the reference convective coefficient obtained from $w_{ref}$. We considered $h_T$ = ${265}\,\hbox {W m}^{-2}\hbox { K}^{-1}$ for low blood flow where $w = {3}\,\hbox {cm s}^{-1}$.

### Convergence analysis

**Fig. 2:**
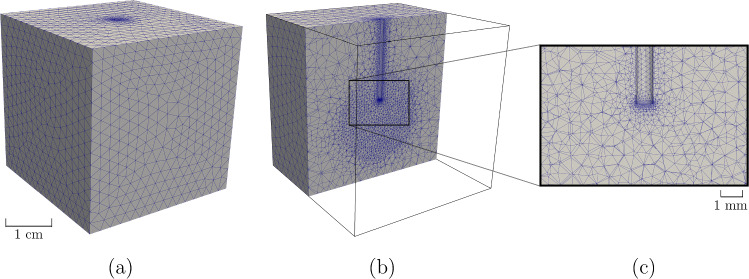
**a** Domain discretization. **b** Spherical mesh refinement around the laser applicator. **c** Zoom in into the laser fiber tip area

An idealized cardiac domain was discretized via tetrahedral finite elements, consisting of 180 737 elements and 30 111 nodes. Progressive mesh refinement was performed within a spherical volume surrounding the laser applicator, with a maximum element size of ${1\times 10^{-3}}\,\hbox {m}$ and a minimum element size of ${4.6\times 10^{-4}}\,\hbox {m}$, where the most significant laser–tissue interaction is expected (see Fig. 2). A subsequent refinement, consisting of a maximum element size of ${6.5\times 10^{-5}}\,\hbox {m}$ and a minimum element size of ${1\times 10^{-5}}\,\hbox {m}$ (the typical size of a cardiac cell), was further imposed closer to the laser tip to ensure highest accuracy where the steepest thermal gradients are generated.

Numerical convergence was conducted for the three thermal models–Fourier, GF, and DPL–to ensure the computational comparison was independent of the numerical approximation. Key elements included temperature and thermal volume damage, guiding us toward an optimal balance between computational efficiency and numerical accuracy.

**Fig. 3:**
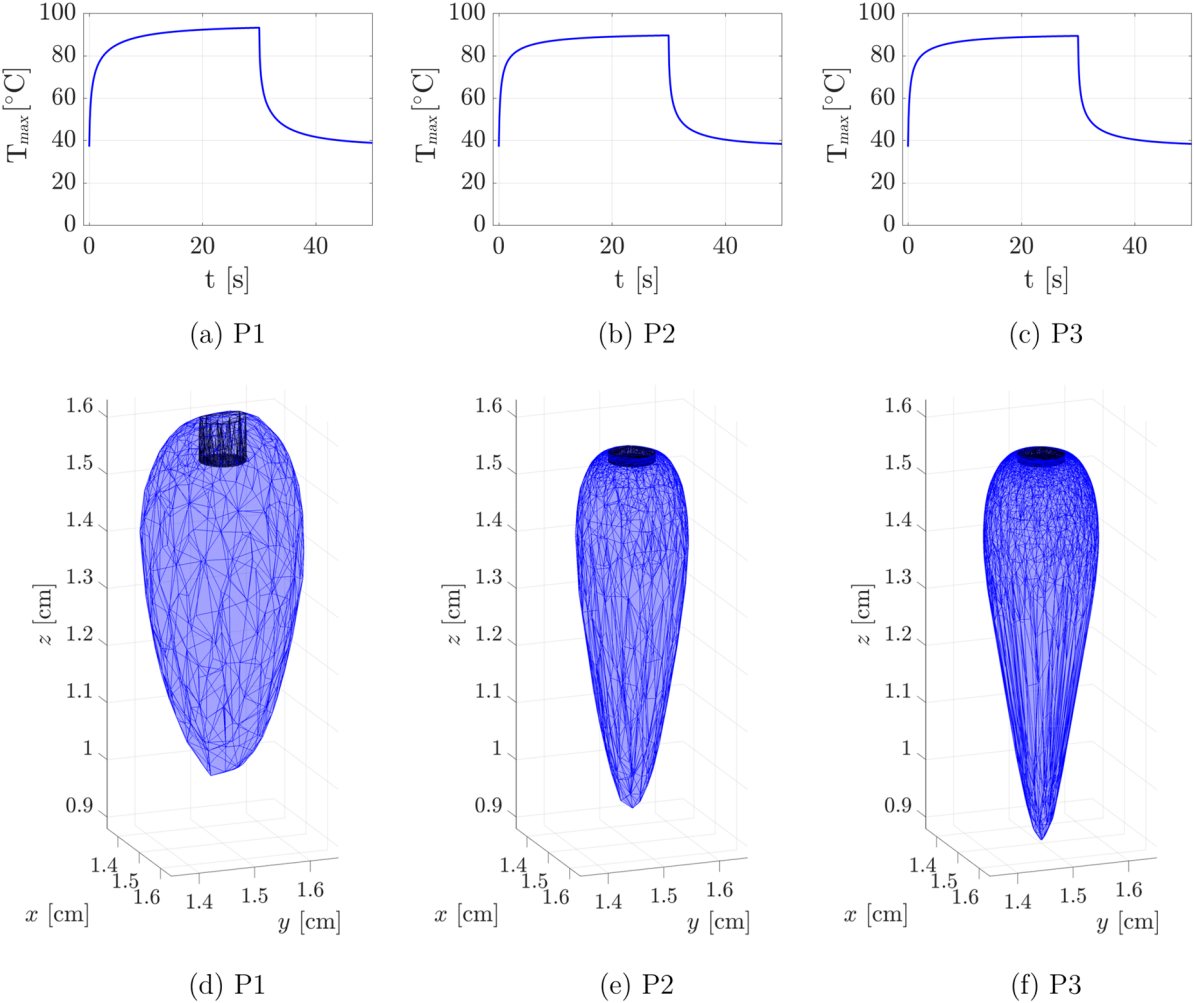
Convergence analysis on DPL model. The upper panels show the maximum temperature $\hbox {T}_{max}$ evolution over time for P1 (**a**), P2 (**b**) and P3 (**c**) elements. In the bottom panels, the thermal volume damage at t = 30 s for P1 (**d**), P2 (**e**), and P3 (**f**) elements is displayed

To explain our convergence analysis, we considered peak temperature and volume lesion dimension (both within the ${50}\,^{\circ }\hbox {C}$ isotherm contours) as key indicators at the end of the ablation process (t = 30 s). We proceeded then to vary the polynomial interpolation degrees from P1 (linear) to P2 (quadratic) to P3 (cubic). Figure 3 shows the DPL peak temperature evolution over time, and the associated thermal damaged volume at t = 30 s, for the three interpolation degrees. Complete information about peak temperature and damaged volumes is provided in Appendix B.

Considering the thermal damage with P1 element, it is evident that the temperature increase significantly affects the area directly below the laser applicator. However, it also considerably impacts the surrounding region near the laser applicator tip. This behavior is unphysical because, as demonstrated in Sect. 5, the radiance—being the primary driver of tissue heating—is almost exclusively concentrated in the area directly below the tip of the laser applicator.

Table 2 summarizes the convergence analysis results for the Fourier, GF, and DPL models. For each indicator and interpolation degree, the percentage error is evaluated with respect to the P3 values, as reference.

Our convergence analysis shows that increasing the interpolation degree from P1 to P2 and P3 results in significant differences in both the peak temperature value and the thermal damage volume. Specifically, the P1 model overestimates both the maximum temperature and the volume of thermal damage compared to the P2 and P3 models, indicating that linear interpolation is not accurate enough to capture the complexity of heat transfer in cardiac tissue with our mesh. Considering an error below 1$\%$, we opted to perform our numerical analysis with P2 elements, balancing the computational cost and the accuracy of the outcomes.

**Table 2:** Convergence analysis indicators: maximum temperature $T_\text {max}$ and thermal damage volume $V_{d}$ (T $\,>\,$ ${50}\,^{\circ }\hbox {C}$) at the end of the ablation procedure at t = 30 s

		Fourier	$\hbox {e}_{\%}$	GF	$\hbox {e}_{\%}$	DPL	$\hbox {e}_{\%}$
$T_\text {max}$ [$\,^{\circ }\hbox {C}$]	P1	93.307	4.319	93.134	4.250	93.754	4.452
	P2	89.476	0.036	89.374	0.041	89.746	0.013
	P3	89.444		89.337		89.758	
$V_{d}$ [$\hbox {mm}^{3}$]	P1	22.492	116.8	21.975	116.05	23.991	115.44
	P2	10.289	0.8	10.106	0.6	10.829	0.8
	P3	10.372		10.171		10.914	

The percentage error is evaluated by comparing the P1–P3 and P2–P3 values

## Results

We employed *Paraview* ^®^*5.11.1* combined with MATLAB for the numerical post-processing of our computational exploration. All simulations follow a protocol of 30 s of ablation at 3 W of applied power. A post-ablation period continues without any active energy source for 5 m, reaching steady-state for temperature and cell state variables.

### Radiance

**Fig. 4:**
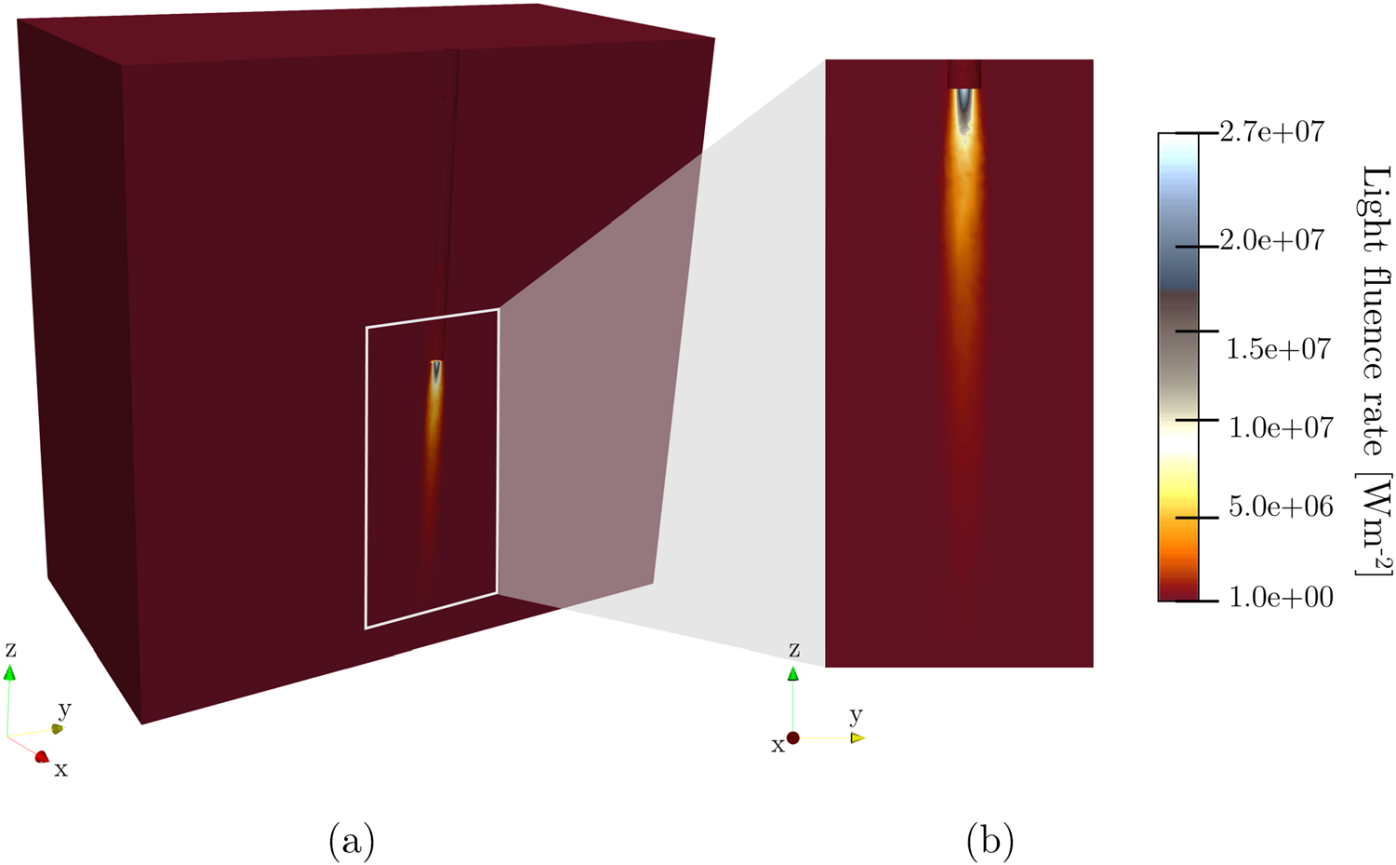
**a** 3D view and **b** z-y zoom-in of light fluence rate distribution

Figure 4 provides outcomes of the light fluence rate $\varphi (x,y,z)$ (1) to investigate the impact of power values on ablation scenarios. The analysis focuses on the spatial distribution of the laser light fluence rate, which is extremely collimated; it penetrates the cardiac tissue sample and mainly propagates along the z-direction. The maximum value of ${2.7 \times 10^{7}}\hbox {W m}^{-2}$ appears on the laser applicator tip.

**Fig. 5:**
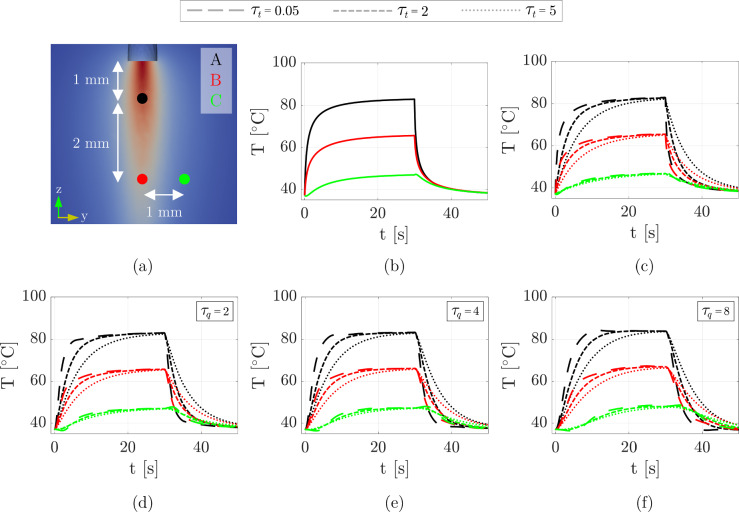
Time constant parametric analysis. **a** A, B, and C: selected points close to the laser beam. **b** Temperature evolutions of the Fourier model. **c** GF case shows multiple traces for $\tau _{t}=0.05,2,5$  s. **d**, **e**, **f** Temperature evolutions of DPL model combining $\tau _{t}=0.05,2,5$  s and $\tau _{q}=2,4,8$  s

### Time constant analysis

We performed a parametric analysis considering $\tau _{q}$ and $\tau _{t}$ as critical quantities that govern the temperature evolution in the GF and DPL models, respectively. Figure 5 highlights the time course of the temperature field for three selected points: A, B, and C in Fig 5a where A is 1 mm away from the laser tip; B is placed on the same z-direction with respect to A but 3 mm away from the laser surface; and point C is at the same elevation of B and 1 mm apart in the y-direction. A comparison of the three formulations is further provided (Fourier (b), GF (c), DPL (d-f)). A common trend is observed: Temperature rises and reaches the peak value at the end of the laser deposition (t = 30 s) and then quickly drops to a rest value in about 20 s for all the models. For the GF formulation, three specific cases are shown considering $\tau _{t}=0.05, 2, 5$  s (Liu and Chen 2010; Zhou et al. 2009). Concerning the DPL model, we performed an additional nine computational experiments, combining the aforementioned value of $\tau _{t}$ and three values of $\tau _{q}$, namely (2, 4, 8  s), following (Sahoo et al. 2014). The closer a point is to the laser applicator (e. g., point A), the higher the peak temperature, which is expected. However, the models differ in how they handle the cooling phase. The DPL model, especially with larger $\tau _{q}$ values, shows a more prolonged cooling phase at greater distances, which may reflect more realistic tissue behavior, as heat diffusion is not instantaneous. The Fourier response is immediate, reflecting the assumption of infinite heat propagation speed, leading to high peak temperatures across all points.

The GF model, as $\tau _{t}$ increases, shows a hump before the plateau phase becoming progressively less pronounced, resulting in a smoother transition to the steady-state temperature at the end of the ablation phase (t = 30 s). Conversely, as $\tau _{t}$ decreases, this hump becomes more pronounced, reflecting a sharper and more localized transient response. In the limiting case where $\tau _{t}=0$, the evolution mirrors the classical Fourier behavior.

In the DPL model, the combination of $\tau _{t}$ and $\tau _{q}$ introduces further complexity. As shown in panels (d-f), both the peak temperature and the cooling phase are significantly affected by the lag times. At higher $\tau _{q}$ values, the peak temperature increases at the end of the ablation phase. For instance, focusing on point A with $\tau _{t}$ = 2, the temperature peaks are ${82.9}\,^{\circ }\hbox {C}$, ${83.1}\,^{\circ }\hbox {C}$, ${83.8}\,^{\circ }\hbox {C}$ for $\tau _{q}$ = 2, 4, 8, respectively. This behavior highlights the model’s capability to incorporate delayed thermal flux dynamics and account for the progressive build-up of temperature gradients over time, mimicking a slower heat dissipation.

### Anisotropy analysis

**Fig. 6:**
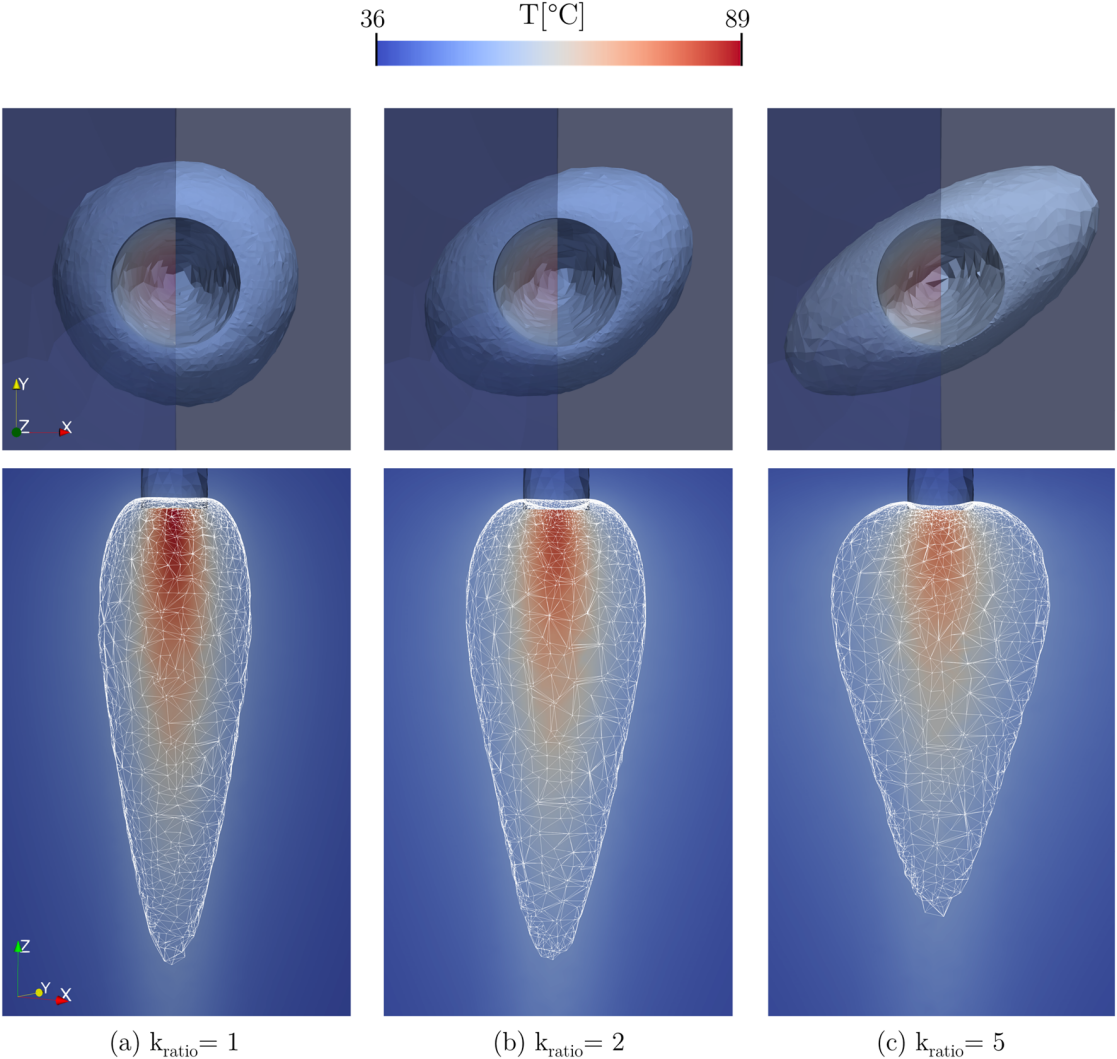
Effect of anisotropy ratio $\textrm{k}_{\textrm{ratio}}$ on temperature distribution and lesion shape considering the ${50}\,^{\circ }\hbox {C}$ isocontour for the DPL model, fixing $\tau _{q} = {4}\,s$ and $\tau _{t} = {2}\,s$. (Top) xy view, (Bottom) zx plane views of the thermal damage lesion

The following analysis characterizes the degree of tissue anisotropy (6) on the optical–thermal response of the myocardium. We performed three different simulations for each model, considering the anisotropy ratio $\textrm{k}_{\textrm{ratio}} =$ 1, 2, 5, to assess the anisotropic sensitivity of the hyperthermic treatment (Molinari et al. 2022). Numerical results demonstrate that the different formulations provide similar values for the ablated volumes; changes are observed by varying the anisotropy factor. Figure 6 shows the thermal volume damage considering temperature above ${50}\,^{\circ }\hbox {C}$ for the DPL model in the case of isotropy (a) and comparing it with anisotropy (b, c). In the latter, a significative spatial curling is observed. Importantly, and in agreement with experimental evidence (Molinari et al. 2022), for higher anisotropy ratios, we observe a general decrease in the thermal damage volume in the z-direction and an increase along the orthogonal plane. Therefore, following thermal anisotropy, a smaller amount of heat is dissipated in depth, which remains stagnant in the proximity of the laser tip fiber.

Analyses conducted for the Fourier and GF formulations are provided in Appendix C.

### Thermal damage

**Fig. 7:**
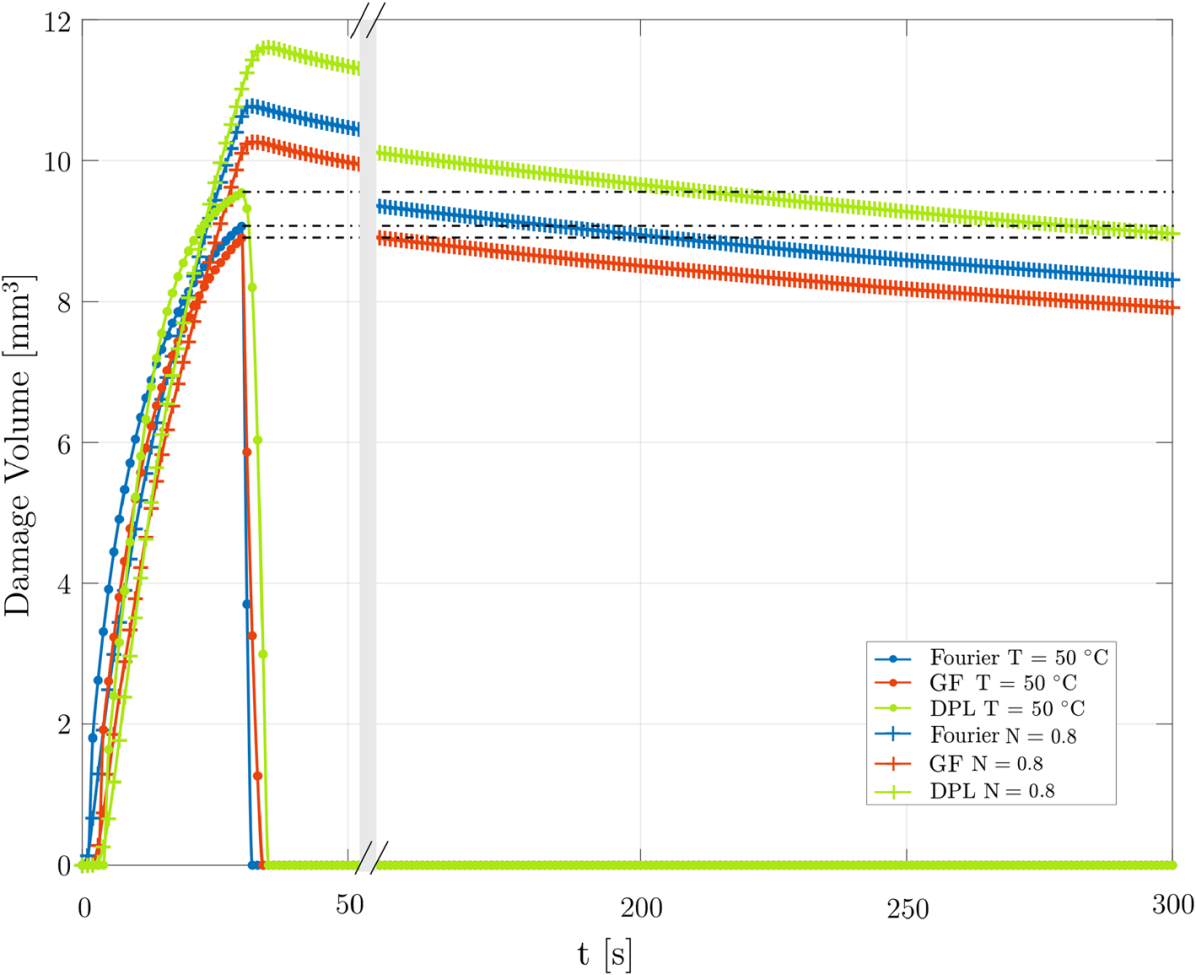
Thermal damage volume over time with the Fourier, GF, and DPL models taking into account the T = ${50}\,^{\circ }\hbox {C}$ and N = 0.8 thresholds

A deeper examination of the ablation-induced lesion is conducted by considering the different heat transfer models. Concerning the three-state cell death dynamics, the native cellular state, represented by N, becomes our guiding reference, assuming that the lesion happens below the selected threshold N $\le$ 0.8, standing as irreversible damage. Figure 7 presents a detailed comparison over five minutes of damage volume evolution comparing the cell death threshold with the classical ${50}\,^{\circ }\hbox {C}$ isotherm. In the case of T = ${50}\,^{\circ }\hbox {C}$, the damage follows the dynamics of the thermal field: a quick rise, a maximum value, and a drop concurrently to the switch off of the laser source. The maximum damage volumes are 9.1$\hbox { mm}^{3}$, 8.9$\hbox { mm}^{3}$, and 9.5$\hbox { mm}^{3}$ for the Fourier, GF, and DPL models, respectively. Conversely, the values indicated by the cellular dynamics result higher at the end of the ablation. However, they recover during the relaxation phase, i.e., the volume decreases, matching the ${50}\,^{\circ }\hbox {C}$ damage volume value at 184.5, 151.4, and 214.3  s for the Fourier, GF, and DPL models, respectively.

Table 3 highlights the percentage thermal volume damage change, $\hbox {V}_{\%}$, considering the reference volume $\hbox {V}_{ref}$ as the volume value at the end of the relaxation phase (t = 300 s) of the cellular dynamics. V$_{\%}$ is calculated considering the damage volume with the T = ${50}\,^{\circ }\hbox {C}$ isotherm at t = 30 s with respect to V$_{ref}.$ As shown in Fig. 7, in the relaxation phase, the volume evolution with N = 0.8 slowly decrease.

**Fig. 8:**
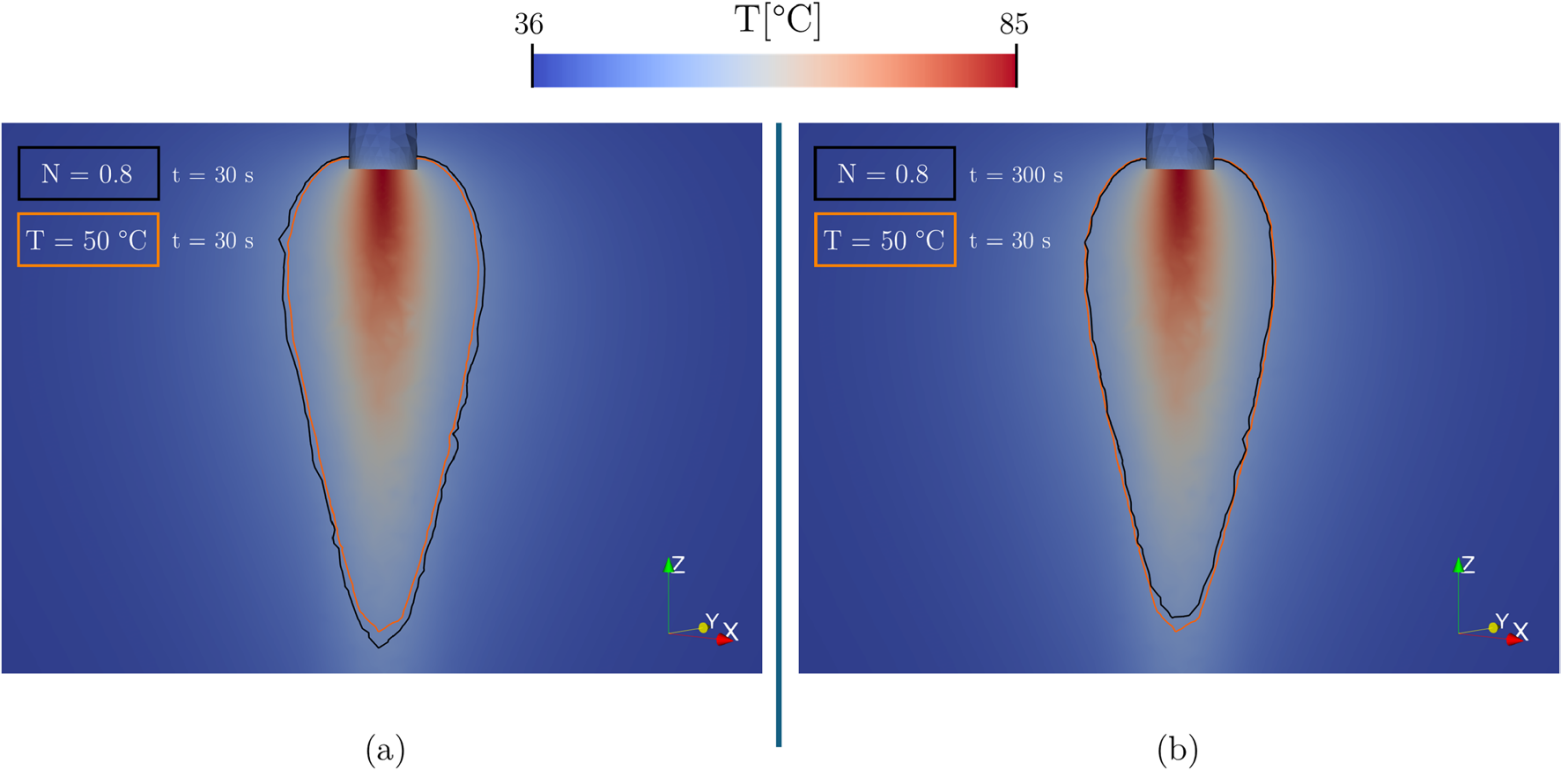
Comparison of computational thermal damage contours of cardiac tissue computed from the DPL model, setting $\textrm{k}_{\textrm{ratio}}$ = 2, $\tau _{q}$ = 4 s and $\tau _{t}$ = 2 s. **a** Comparison between V$_{\textrm{N} = 0.8}$ and V$_{\textrm{T} = {50}\,^{\circ }\hbox {C}}$ at the end of ablation (t = 30 s). **b** Comparison between V$_{\textrm{N} = 0.8}$ at the end of the relaxation phase (t = 300 s) and V$_{\textrm{T} = {50}\,^{\circ }\hbox {C}}$ at the end of ablation (t = 30 s)

By way of example, in the thermal damage of the DPL model (Fig. 8), there is a trend inversion: At the end of the ablation phase (Fig. 8a), the V$_{\textrm{N} = 0.8}$ is higher than V$_{\textrm{T} = {50}\,^{\circ }\hbox {C}}$, whereas the opposite situation appears considering V$_{\textrm{T} = {50}\,^{\circ }\hbox {C}}$ at t = 30 s and V$_{\textrm{N} = 0.8}$ at t = 300 s (Fig. 8b).

**Table 3:** Thermal damage volume change. V$_{ref}$ is the volume damage considering the three-state threshold N = 0.8 at t = 300 s

	Fourier	GF	DPL
V$_{ref}$ [$\hbox {mm}^{3}$] (N = 0.8)	8.3077	7.9147	8.9575
V$_{\%}$ (V$_{\textrm{T} = {50}\,^{\circ }\hbox {C}}$ vs V$_{ref}$)	+9.1 $\%$	+12.4 $\%$	+6.5 $\%$

The volume percentage change V$_{\%}$ is calculated considering the damage volume of T = ${50}\,^{\circ }\hbox {C}$ isotherm at t = 30 s with respect to V$_{ref}$

**Fig. 9:**
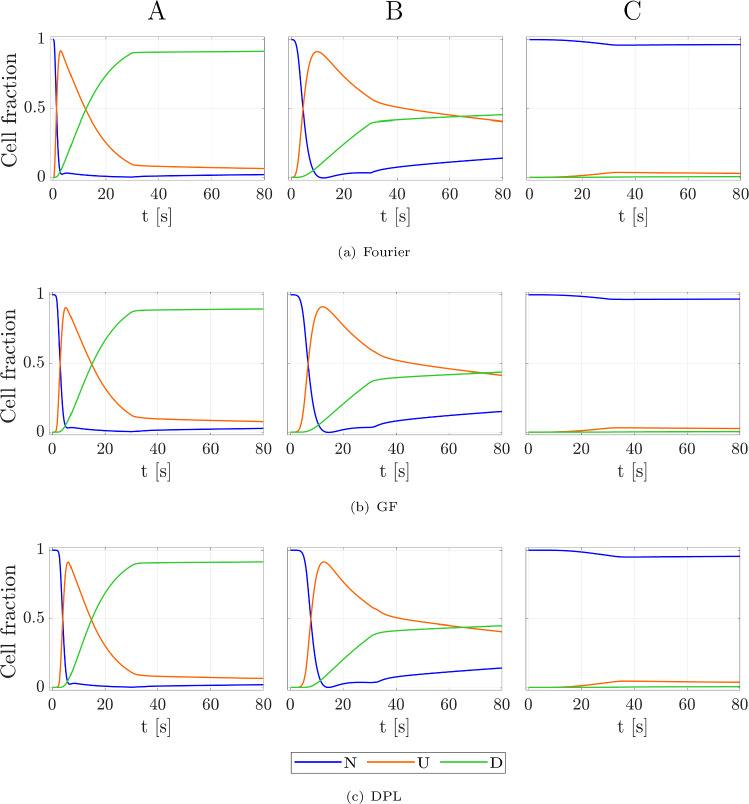
Three-state cell dynamics during an ablation hyperthermic protocol treatment of 30 s of ablation: native (N), unfold (U), and damage (D) states for the Fourier **a**, GF **b**, and DPL **c** models for the main focused A, B, and C points, close to the laser applicator

We conclude discussing the temporal evolution of N, U, and D variables. Figure 9 shows the time course of the three state variables, referring to the Fourier (a), GF (b), and DPL (c) models, evaluated in three specific cardiac domain points (see Fig. 5a). We observed the state cell evolution over five minutes of simulation until an asymptotical behavior occurred. The common initial condition is that all cells are in the native state, namely $\textrm{N}$ = 1, $\textrm{U}$ = 0, and $\textrm{D}$ = 0. As depicted in the figure, especially for points A and B, there is a temporal shift from the Fourier to DPL models for N, U, and D. This happens because the GF and DPL models are governed by time lag constants that control the general dynamics of the temperature distributions within the myocardium domain.

## Conclusions and discussions

The present study proposes a finite element computational model of laser ablation on an idealized cardiac domain. The optical–thermal coupled formulation involves the ODA model to describe the laser light propagation in the myocardium and the Fourier, GF, and DPL thermal theories to simulate bioheat transfer. The coupled multiscale framework also integrates three-state cell death dynamics to explore the spatiotemporal damage phenomenology occurring during LA procedures. The numerical investigation identified the most significant factors governing cardiac LA as a possible treatment. At first, the spatial distribution of light fluence during power application directly influences the depth and shape of tissue damage. Accordingly, precise modulation of laser power could enhance ablation efficacy, minimizing damage to the surrounding healthy tissue. This observation aligns with existing literature, where precision in power delivery is emphasized as a key factor in effective cardiac ablation procedures (Zhang et al. 2023; Kabiri and Talaee 2021). The development of more refined laser power modulation techniques includes in silico-based machine learning algorithms to maintain optimal ablation conditions, improving the precision and safety of the procedure.

Second, the time constant analysis governing the temperature evolution in higher-order models was revealed to be critical in view of future uncertainty quantification studies (Brandstaeter et al. 2021). The temperature profile, which peaks at the end of laser deposition before returning to the baseline, underscores the importance of timing in the ablation process. The literature often highlights the significance of controlling the heating and cooling phases to prevent collateral damage (Molinari et al. 2022). Our results support such evidence by demonstrating the effect of different time constants on post-ablation tissue recovery.

Finally, a material anisotropy analysis confirms that microstructural features significantly alter the extent and shape of thermal damage also in higher-order formulations. Such a result is particularly interesting given the intrinsic biological variability requiring a priori patient-specific analyses. Current scientific discussions often point out the need for personalized approaches in cardiac ablation (Sang and Ye 2023), and the present study provides empirical support for tailoring procedures based on tissue characteristics. The observed impact of tissue anisotropy suggests that ablation strategies should be customized, thus involving pre-procedure imaging to assess tissue properties and adjust the laser parameters accordingly.

Limitations to our study need to be acknowledged. First, we used an idealized 3D computational domain that does not account for the complex boundaries and heterogeneity of a cardiac ventricular chamber. Generalizations of the results require further investigation, followed by using patient-specific anatomical models to improve clinical relevance. Moreover, material parameters lack dedicated experimental characterization, considering the few studies addressing *ex vivo* cardiac tissue ablation. We hope that future experimental studies support the validation of higher-order thermal models, thus improving their predictability in clinical scenarios. Lastly, the laser source in this study was modeled with uniform power delivery, which may not reflect real-world conditions where deviations in laser energy could affect treatment outcomes. We foresee incorporating dynamic feedback to adapt the laser application in real-time also considering additional heat relaxation time effects (Singh and Melnik 2019).

In conclusion, this investigation emphasizes the potential role of innovative modeling techniques, highlighting crucial aspects of using different theories to improve the precision and effectiveness of cardiac LA procedures. The higher-order DPL model is the most comprehensive framework for accurately describing what occurs in cardiac biological samples, paving the way for optimizing clinical outcomes in cardiac ablation procedures by deepening our understanding of laser–tissue interactions. Ultimately, the findings contribute to developing standardized protocols and guidelines that can be effectively implemented in cardiac electrophysiology practice.

## Figures and Tables

**Fig. 10:**
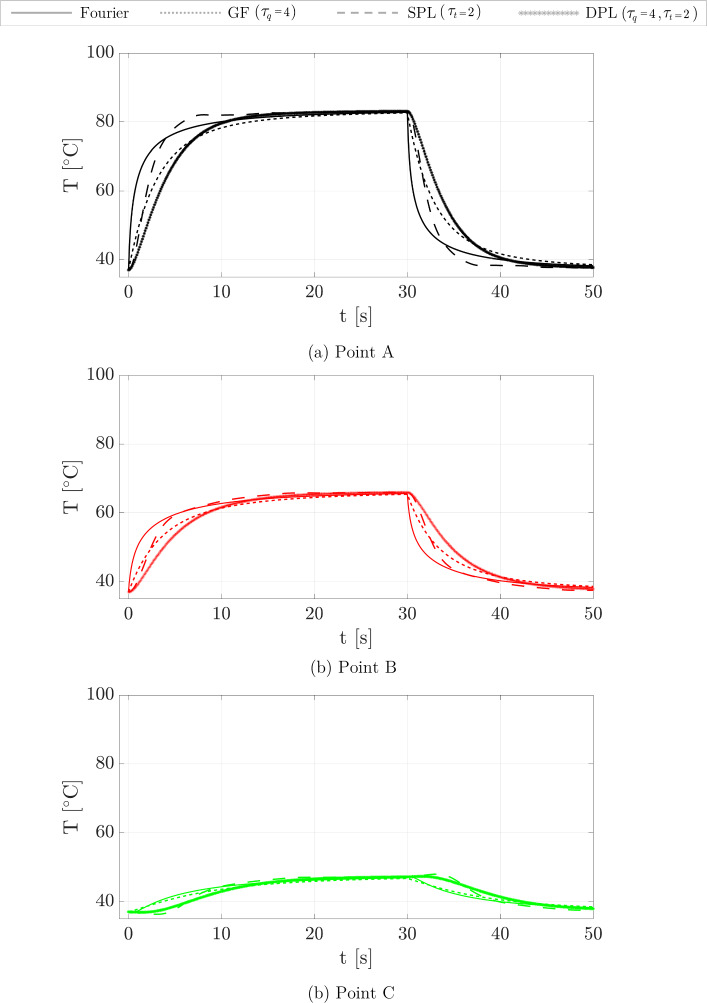
Temperature evolutions for A **a**, B **b**, and C **c** points considering the Fourier, GF with $\tau _t={2}\,s$, SPL with $\tau _q={4}\,s,$ and DPL with $\tau _q={4}\,s$ and $\tau _t={2}\,s$ models

**Fig. 11:**
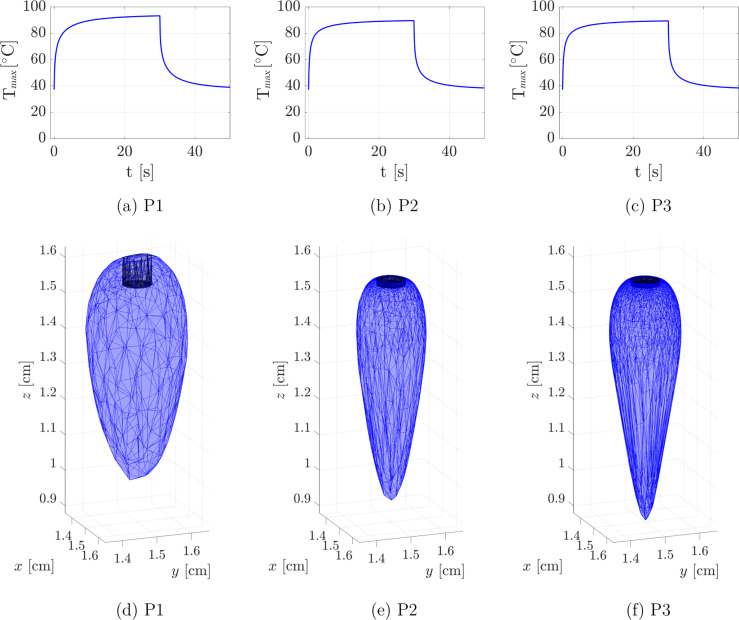
Convergence analysis on Fourier model. The upper panel shows the maximum temperature T$_{max}$ evolution over time for P1 **a**, P2 **b**, and P3 **c** elements. In the bottom panel are displayed the thermal volume damage at t = 30 s for P1 **d**, P2 **e**, and P3 **f** elements

**Fig. 12:**
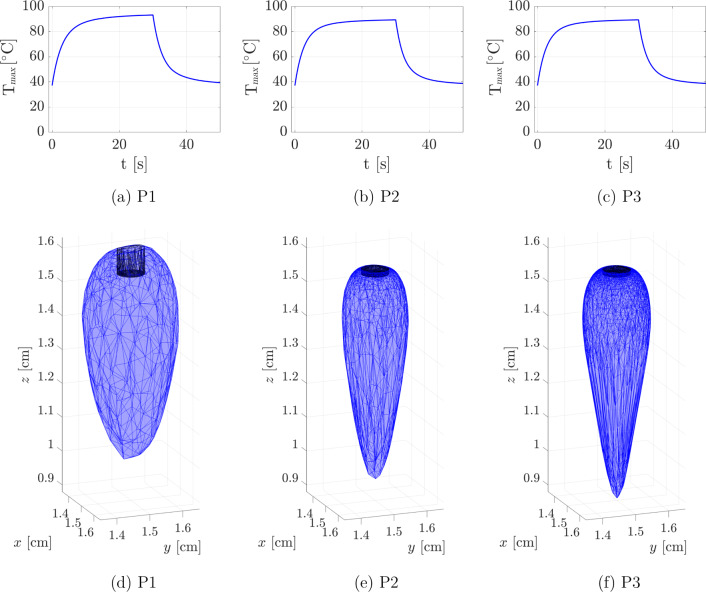
Convergence analysis on GF model. The upper panel shows the maximum temperature T$_{max}$ evolution over time for P1 **a**, P2 **b**, and P3 **c** elements. In the bottom panel are displayed the thermal volume damage at t = 30 s for P1 **d**, P2 **e**, and P3 **f** elements

**Fig. 13:**
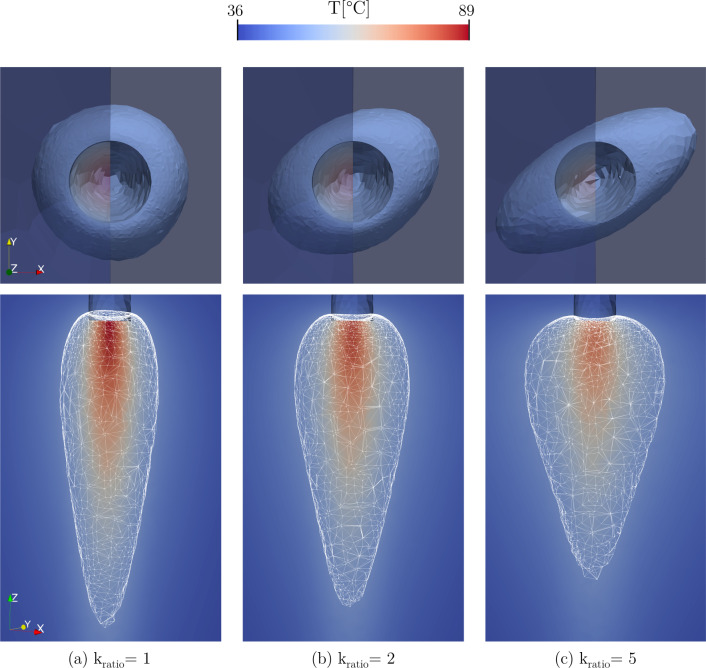
Effect of anisotropy ratio $\textrm{k}_{\textrm{ratio}}$ on temperature distribution and lesion shape considering the ${50}\,^{\circ }\hbox {C}$ isocontour for the Fourier model. (Top) xy view of the thermal damage lesion; (Bottom) zx plane of temperature distribution

**Fig. 14:**
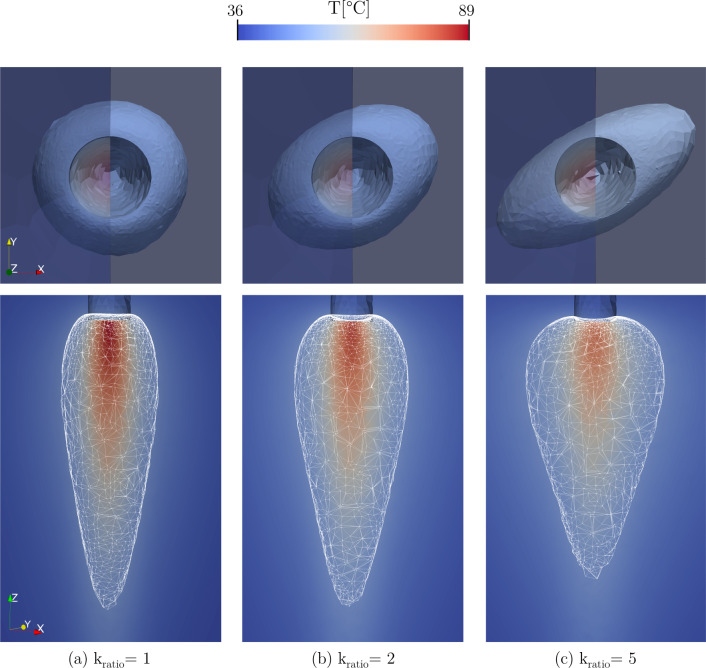
Effect of anisotropy ratio $\textrm{k}_{\textrm{ratio}}$ on temperature distribution and lesion shape considering the ${50}\,^{\circ }\hbox {C}$ isocontour for the GF model, fixing $\tau _{t}$ = 2 s. (Top) xy top view of the thermal damage lesion; (Bottom) zx plane of temperature distribution

## Data Availability

No datasets were generated or analysed during the current study.

## Appendix A SPL model

For the sake of completeness, we developed the single-phase lag (SPL) model to highlight the potential differences compared to the generalized Fourier-based model (GF) model. This allowed us to better understand how the introduction of the phase lag in the heat flux, as proposed in the SPL model, influences thermal behavior, providing a broader perspective on the progression of heat transfer models. The SPL is mathematically expressed as:

A1 $$\begin{aligned} &  \tau _{\textrm{q}} {\rho } \mathrm {c(T)} \frac{\partial ^2 \textrm{T}}{\partial \textrm{t}^{2}} + {\rho } \mathrm {c(T)}\frac{\partial \textrm{T}}{\partial t}\nonumber \\ &  \quad =\nabla \cdot (\textbf{k}\mathrm {(T)} \nabla \textrm{T}) + \textrm{Q}_{\textrm{b}} + \textrm{Q}_{\textrm{m}} + \textrm{Q}_{\textrm{s}} \end{aligned}$$

Figure 10 shows the temperature dynamic comparisons between the Fourier, GF, SPL, and DPL models, for three selected points (see Fig. 5a).

## Appendix B convergence analysis

Concerning the convergence analysis, Figs. 11 and 12 depict the maximum temperature evolution over time in panels (a), (b), and (c) considering the P1, P2, and P3 element degrees, for the Fourier and GF models, respectively. In the same way, panels (d), (e), and (f) show the thermal volume damage at the end of the ablation process (t = 30 s).

## Appendix C anisotropy

Figures 13 and 14 illustrate the effect of the anisotropy cardiac tissue degree on the optical–thermal response of the myocardium, considering the Fourier and GF heat models, respectively. We explored the anisotropy ratio $\textrm{k}_{\textrm{ratio}} =$ 1, 2, and 5. In both figures, panels (a) regard the isotropic case. Increasing the value of $\textrm{k}_{\textrm{ratio}}$ from 2 (b) to 5 (c), the temperature distribution shape mutates. In the upper panels, the higher the value of $\textrm{k}_{\textrm{ratio}}$, the higher the elliptical shape of the damage volume. Furthermore, for higher anisotropy ratios, we observe a general decrease in the thermal damage volume in the z-direction and an increase along the x-direction. Therefore, following thermal anisotropy, a smaller amount of heat is dissipated in depth which remains stagnant in the proximity of the laser tip fiber.
